# Editorial: Gender Roles in the Future? Theoretical Foundations and Future Research Directions

**DOI:** 10.3389/fpsyg.2019.01965

**Published:** 2019-09-04

**Authors:** Alice H. Eagly, Sabine Sczesny

**Affiliations:** ^1^Department of Psychology, Northwestern University, Evanston, IL, United States; ^2^Department of Psychology, University of Bern, Bern, Switzerland

**Keywords:** gender prejudice, social role theory, communion, agency, gender stereotypes, gender roles

The study of gender has become a major focus of research in psychology and in social psychology in particular. Among early contributors to this study, Eagly (1987) formulated social role theory to explain the behavior of women and men as well as the stereotypes, attitudes, and ideologies that are relevant to sex and gender. Enhanced by several extensions over the intervening years, this theory became a pre-eminent theory of gender in social psychology (Eagly and Wood, 2012). Also, over the last decades, social psychologists have developed a variety of related approaches to understanding gender, including, for instance, theories devoted to stereotype threat, status, backlash, lack of fit to occupational roles, social identity, and categorization. The conference that preceded this Research Topic, sponsored by the European Association of Social Psychology and the Society for Personality and Social Psychology, featured work that fit within the broad umbrella of social role theory and related approaches.

The contemporary interest in the psychology of gender reflects its centrality in the understanding of social behavior. Gender continues to be a driving force in world politics and economics, as evident in the struggles of women to attain parity in political and economic institutions, the transformative impact of the #me-too movement, and the falling birthrates in many nations as women opt for careers instead of large families. In addition, binary gender itself is facing challenge as the two primary sex categories of female and male yield to accommodate multiple gender and sexual identities, including non-binary identities and transgender status.

One of the central topics of the social psychology of gender is gender stereotypes, understood as consensual beliefs about the attributes of women and men. Although describing the content of gender stereotypes might seem to be a task already accomplished many decades ago (e.g., Broverman et al., 1972), research on this matter has continually expanded. Not only has recent research described change in gender stereotypes over time (Eagly et al., 2019), but also this Research Topic includes the Hentschel et al. article that identifies facets underlying these stereotypes' two primary dimensions of agency and communion. Their analysis of agency thus reveals the facets of independence, instrumental competence, and leadership competence and of communion yields the facets of concern for others, sociability, and emotional sensitivity. Other advances in stereotype research consider intersectionalities between gender and other social attributes as well as the prescriptive aspect of gender stereotypes by which they define what members of each sex should and should not do. Illustrating these advances, Koenig's research explores prescriptive stereotypes for the intersections of gender with age from toddlerhood to old age. Among her findings is a weakening of these gender stereotypes in relation to elderly women and men.

Gender stereotypes exert influence in daily life even when they compete with the influences of other social roles. In particular, occupational roles have demands that may be more or less consistent with gender roles. In extending social role theory to account for such circumstances, Eagly and Karau (2002) argued that the female gender stereotype is generally inconsistent with leader roles because of the expectations that women are communal and that leaders, like men, are agentic. Consequently, women can suffer discrimination in relation to leadership roles because many people believe that they are insufficiently agentic to perform effectively as leaders. Manzi raises the issue of whether parallel discriminatory processes exist for men who occupy or seek to occupy roles with primarily communal demands. The article by Block et al. further addresses men's occupancy of communal roles by analyzing the low representation of men in healthcare, early education, and domestic (HEED) roles. Their research shows that, consistent with gender stereotypes, men tend to have agentic values that focus on status, competition, and wealth and thus are not attracted to careers with a focus on caring for others. However, as Van Grootel et al. demonstrate, men tend to underestimate the extent to which other men approve of men's communal traits and behaviors. Correction of this pluralistic ignorance fosters men's greater endorsement of communal values and support for progressive gender-related social change. In a different demonstration of how to reduce the power of existing gender stereotypes, Olsson and Martiny review research on exposure to counterstereotypical role models. They conclude that such exposures do hold promise for promoting counterstereotpical goals and aspirations, especially in girls and women.

For leadership, gender makes a difference, given the definition of leadership primarily in culturally masculine terms that disfavor women. Vial and Napier offer clever demonstrations that people do view agentic traits as more important than communal traits for successful leaders, thus confirming women's disadvantage for attaining leader roles. Communal traits appear to be a nice, but inessential add-on for leaders. Another disadvantage for women, as shown by Player et al., is that male candidates for leadership are valued more highly for their perceived potential to be a good leader rather than their past performance. Female candidates, in contrast, are valued more for their past performance and given relatively little credit for their potential. Consistent with the female stereotype of low agency, women thus have the burden of proving their leadership competence rather than merely being trusted to have potential for the future. As shown by Gruber et al., some women do emerge as leaders, and greater facial attractiveness facilitates their emergence by fostering the ascription of social competence to them. These researchers have yet to investigate the importance of facial attractiveness to male leaders.

Increasing gender diversity in organizations is surely an important social goal for advocates of gender equality. Yet, organizational processes are not so simple that merely adding women catalyzes gains for other women. In fact, women in leadership roles do not necessarily work to change organizational norms to insure equal opportunity for other women, as Sterk et al. argue. Instead, senior women may accept negative stereotypes about women's lesser capacity for leadership. Such “queen bee” senior women may distance themselves from junior women and thus exert negative effects on them. Moreover, as van Dijk and van Engen explain, despite the presence of gender-diverse work groups, organizational behaviors are often constrained by self-reinforcing gender role expectations that perpetuate traditional gender-unfair practices.

Gender stereotypes exert influence in other situations as well. One such setting is high-stakes aptitude tests whose outcomes affect the opportunities of women and men. As shown by the Leiner et al. research on Austrian medical school aptitude tests, there are intriguing sex differences in the ways that female and male test takers perceive the test situation. In particular, the women experienced greater test anxiety than men and perceived the test as less fair. Another realm of social behavior that is fraught with gender issues is sexual coercion and rape. Gravelin et al. provide a thorough review of what is now a large research literature on tendencies to blame the victim of acquaintance rape. Also related to sexual violence is an incident in Germany of mass sexual assault on New Year's Eve of 2015. The discourse that ensued receives careful analysis by Hannover et al. One question that Germans faced is whether the largely Muslim perpetrators of these assaults were motivated by particularly sexist attitudes toward girls and women that emanated from their religion. The findings of this research instead implicated, not a particular religion, but high levels of religiosity and fundamentalism as precursors of the sexist beliefs that fostered violence against women.

In a world in which gender is always in flux, the future of gender relations is uncertain. To help understand this future, Gustafsson Sendén et al. asked Swedes to indicate what they think that the traits of Swedish women and men were in the past, are in the present, and will be in the future. Replicating earlier research by Diekman and Eagly (2000), respondents perceived women to increase in agentic traits over time but remain more communal than men. Such beliefs, derived from the abstract belief that gender equality is increasing, may not reflect actual changes in stereotype content over time (Eagly et al., 2019).

The contemporary challenges to the binary view of sex, gender, and sexuality receive important exploration in the essay by Morgenroth and Ryan. They review earlier writing by the philosopher Judith Butler, who advocated “gender trouble” that would disrupt the binary view of gender. As these authors suggest, Butler's ideas can guide understanding of some of the ways that performance socially constructs gender in society. Butler's writings on performativity and related themes can provide intriguing hypotheses for systematic empirical exploration by social psychologists. In the meantime, other social psychologists argue that the way forward in gender theory entails exploring how gender is and is not socially constructed by producing research that also considers the biological grounding of some patterns of male and female behavior (Eagly and Wood, 2013). From this interactionist perspective, nature, and nurture are intertwined in producing the phenomena of gender.

The articles included in this Research Topic are broadly positioned across the field of social psychology, which encompasses a wide range of themes pertaining to sex and gender. Some of these themes link social psychology to other areas of psychological specialization, such as personality, developmental, cultural, industrial-organizational, and biological psychology as well as to the other social science disciplines of sociology, political science, and economics. In invoking other disciplines and psychology subfields, many of the authors whose work appears in this Research Topic recognize the importance of social roles as a central integrative concept in theories of gender. These articles thereby complement social role theory by reaching out to build an extended theoretical foundation for gender research of the future.

## Author Contributions

Both authors listed have made a substantial, direct and intellectual contribution to the work and approved it for publication.

### Conflict of Interest Statement

The authors declare that the research was conducted in the absence of any commercial or financial relationships that could be construed as a potential conflict of interest.
